# Clinical management and pharmaceutical care of severe *Chlamydia psittaci* Pneumonia complicated with rhabdomyolysis and multiple organ dysfunction: a case report and literature review

**DOI:** 10.3389/fcimb.2026.1785697

**Published:** 2026-05-13

**Authors:** Nan Huang, Jinshuo Fan, Liang Xiong, Haibo Lei, Zheng Liu, Sanlan Wu, Weijing Gong

**Affiliations:** 1 Clinical pharmacy, The Central Hospital of Xiangtan (The affiliated hospital of Hunan university), Xiangtan, China; 2 Department of respiratory and critical care medicine, Union Hospital, Tongji Medical College, Huazhong University of Science and Technology, Wuhan, China; 3 Department of Pharmacy, Union Hospital, Tongji Medical College, Huazhong University of Science and Technology, Wuhan, China

**Keywords:** *Chlamydia psittaci*, multiple organ dysfunction, pharmaceutical intervention, severe pneumonia, targeted next-generation sequencing (TNGS)

## Abstract

Psittacosis, also known as “bird fever”, is a zoonotic disease caused by *Chlamydia psittaci* (*Cps*) infection. Its clinical manifestations are similar to those of many respiratory tract infections and lack specificity, leading to a high risk of missed diagnosis or misdiagnosis, which poses great challenges to clinical treatment. With the advancement of diagnostic techniques, timely detection using targeted next-generation sequencing (tNGS) can provide a reliable basis for the early clinical diagnosis of *Cps* infection and the formulation of individualized treatment regimens. This paper reports a severe case of *Cps* pneumonia complicated with multiple organ injuries including acute respiratory failure, rhabdomyolysis, and hepatorenal dysfunction. Clinical pharmacists were involved in treatment regimen optimization, medication monitoring, and patient education. Based on the confirmed diagnosis by tNGS, an individualized anti-infective strategy was formulated according to the pathogen characteristics and the patient’s complications, with simultaneous monitoring of multiple organ functions and adverse drug reactions. This case provides clinical evidence for the individualized treatment of severe *Cps* pneumonia with multiple organ injuries, and also confirms the critical role of tNGS in the diagnosis of rare pathogens.

## 1 Introduction

*Cps* is an obligate intracellular Gram-negative bacterium characterized by a unique biphasic developmental cycle. The prevalence of *Cps* infection is relatively high in avian species worldwide, with a reported positivity rate of 3.19% (18/564; 95% CI 2.03–4.99%) (Sukon et al., 2021; Kasimov et al., 2022). As a zoonotic pathogen, *Cps* is associated with a wide range of infections in both wild and domestic animals, particularly birds. Direct contact with infected birds and inhalation of contaminated airborne droplets are the primary risk factors for transmission. Additional risk factors include pregnancy, overcrowding, exposure to avian excreta, and close contact with cats and dogs (Wang et al., 2024b). Furthermore, *Cps* has demonstrated the potential to evolve human-to-human transmission through multiple routes (Zhang et al., 2022). In humans, *Cps* infection can induce influenza-like symptoms, pneumonia, endocarditis, fever, chills, myalgia, and headache. Notably, it may also increase susceptibility to co-infection with other respiratory pathogens (Halánová et al., 2019; Yehia et al., 2023; Ghasemian et al., 2024). Due to its non-specific clinical presentation, human *Cps* infection is frequently misdiagnosed as community-acquired pneumonia (CAP). Most CAP cases are treated empirically without targeted diagnostic testing, which often leads to progression to severe community-acquired pneumonia (SCAP) and poses significant challenges to clinical management. Therefore, timely and accurate identification of the pathogen is crucial (Hogerwerf et al., 2017; Qu et al., 2022).

In recent years, the advancement of next-generation sequencing (NGS) technologies has gradually overcome the limitations of traditional diagnostic methods, leading to a substantial increase in their clinical application. The two most commonly used approaches are metagenomic next-generation sequencing (mNGS) and tNGS (Rodino and Simner, 2024). mNGS has been validated as a robust tool for improving pathogen detection rates in patients with SCAP (Wu et al., 2025; Zhang et al., 2025). This unbiased technique enables the simultaneous identification of diverse pathogens, including viruses, bacteria, and fungi, and can even detect rare or uncharacterized microorganisms. Additionally, mNGS exhibits high reliability in terms of negative predictive value, effectively ruling out irrelevant pathogens. It provides accurate diagnostic insights for complex cases with unclear etiologies, thereby enhancing the overall efficiency of clinical diagnosis. mNGS can detect *Cps* within 48 hours, significantly shortening the diagnostic turnaround time and playing a pivotal role in outbreak control and personalized treatment implementation (Li et al., 2021; Tang et al., 2022; Yuan et al., 2024; Wen et al., 2025; Wu et al., 2025). However, the detection sensitivity of mNGS is highly susceptible to interference from host-derived background nucleic acids. In contrast, tNGS employs pre-sequencing nucleic acid capture and enrichment of target pathogens, followed by high-throughput sequencing and bioinformatics analysis. This targeted approach effectively increases the relative abundance of pathogen-derived sequences, thereby improving detection sensitivity, and is recognized as a rapid and cost-effective diagnostic method. Compared with mNGS, tNGS has consistently demonstrated higher detection rates for *Cps* (Yu et al., 2025). Studies have reported that tNGS can successfully detect *Pneumocystis jirovecii* in samples where mNGS yielded negative results (Yin et al., 2024). In genetic analysis, tNGS is also widely used to identify rare variants in known genes (Kristan and Debeljak, 2025). Moreover, tNGS has shown a high positive detection rate for pathogens in respiratory specimens. Notably, it maintains a sensitivity of 70.8–95.0% even in samples with low pathogen loads, including blood and cerebrospinal fluid. tNGS is also effective in detecting drug-resistant *Mycobacterium tuberculosis* strains, as it can identify resistance-conferring genes to guide clinical treatment decisions, thus demonstrating distinct advantages over mNGS in this context (Chen et al., 2024).

The lungs are the most common site of *Cps* infection (McGovern et al., 2021). In addition, *Cps* infection can be complicated by rhabdomyolysis (Matsushima et al., 2002; Takayanagi et al., 2005; Zhang et al., 2022) and may induce multiple organ dysfunction (Meijer et al., 2021; Su et al., 2021; Wu et al., 2025), leading to extremely poor prognosis. Therefore, clinicians must pay close attention to such cases, ensuring early detection and prompt intervention. Herein, we report a case of severe *Cps* pneumonia complicated by rhabdomyolysis and multiple organ dysfunction, which was definitively diagnosed using tNGS.

## 2 Case report

**Figure 1:**
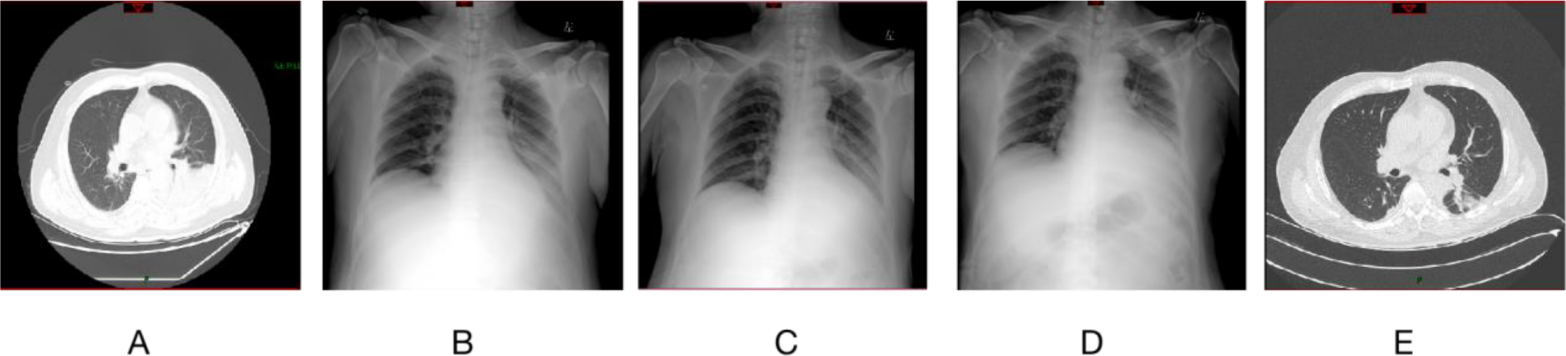
Chest CT and X-ray findings during the patient’s disease course. **(A)** Chest CT on December 2, 2025: A small amount of bilateral pleural effusion (predominantly on the left side), near-complete consolidation of the left lower lung lobe, and scattered subsegmental atelectasis/inflammatory lesions in both lungs. **(B)** Chest X-ray on December 3, 2025: (1) Large patchy opacities in the left lower lung field, consistent with infectious lesions; linear opacities in the right lung field; (2) A small amount of left-sided pleural effusion; (3) The tip of the PICC catheter is located in the inner zone of the left upper lung field. **(C)** Chest X-ray on December 4, 2025: (1) Minimal inflammatory lesions and subsegmental atelectasis in the left lower lung field; slightly decreased translucency in the right lower lung compared with the previous image; (2) A small amount of left-sided pleural effusion, increased compared with the prior study; (3) Cardiomegaly and aortic knob calcification; (4) The tip of the PICC catheter is located in the inner zone of the left upper lung field. **(D)** Chest X-ray on December 5, 2025: (1) Minimal inflammatory lesions and subsegmental atelectasis in the left lower lung field, similar to the previous finding; (2) A small amount of left-sided pleural effusion, unchanged from the prior image; (3) Cardiomegaly and aortic knob calcification; (4) The tip of the PICC catheter is located in the inner zone of the left upper lung field. **(E)** Chest CT on December 10, 2025: (1) Left-sided pleural effusion with consolidation of the left lower lung lobe, overall extent reduced compared with the initial scan; a small amount of right-sided pleural effusion; (2) Minimal subsegmental atelectasis/chronic inflammation in both lungs.

A 61-year-old male patient was admitted to hospital on December 2, 2025, with the chief complaint of fever and cough for 8 days. Eight days prior to admission, he developed fever, cough, and expectoration without obvious predisposing causes, and these symptoms progressively worsened, accompanied by fatigue, anorexia, and gait instability. He suffered a sudden syncope on November 28, 2025. Tests at the local hospital showed the following results: C-reactive protein (CRP) 168 mg/L, alanine aminotransferase (ALT) 66 U/L, creatine kinase (CK) 17323 U/L, lactate dehydrogenase (LDH) 484 U/L, and positive influenza A virus. Chest computed tomography (CT) indicated an infectious lesion in the left lower lung lobe. Despite receiving relevant treatment, his symptoms failed to improve. He visited the emergency department on December 1, 2025, and subsequent examinations revealed elevated indices: CRP 293.20 mg/L↑, ALT 425 U/L↑, aspartate aminotransferase (AST) 1035 U/L↑, CK > 20640 U/L↑, LDH 1708 U/L↑, and creatine kinase-MB (CK-MB) mass 16.6 ng/mL↑. A diagnosis of severe pneumonia and rhabdomyolysis was made. He was given symptomatic treatment and transferred to the Department of Respiratory and Critical Care Medicine, and further admitted to the Respiratory Intensive Care Unit (RICU) on the same day due to respiratory failure. Past medical history: The patient had a history of meningioma surgery in 2018 and essential hypertension, for which he had been regularly taking nifedipine controlled-release tablets for long-term management. He had no history of drug allergy. Physical examination on admission: Temperature 36.3°C, heart rate (HR) 112 beats/min, respiratory rate (RR) 41 breaths/min, blood pressure (BP) 147/96 mmHg, body mass index (BMI) 26.12 kg/m². The patient was conscious; breath sounds in both lungs were slightly coarse, and inspiratory crackles could be auscultated in the anterior upper region of the right lung. No obvious edema was observed in both lower extremities. Auxiliary examinations: Emergency chest CT on December 1, 2025, showed a small amount of bilateral pleural effusion (predominantly on the left side), near-complete consolidation of the left lower lung lobe, and scattered subsegmental atelectasis/inflammatory lesions in both lungs (**Figure 1A**). Re-examination on December 2, 2025, revealed the following levels: CRP 200.64 mg/L↑, ALT 337 U/L↑, AST 528 U/L↑, and procalcitonin (PCT) 1.15 ng/mL↑. Admission diagnoses: 1. Pulmonary infection; 2. Severe pneumonia; 3. Rhabdomyolysis; 4. Respiratory failure; 5. Hepatorenal dysfunction.

## 3 Treatment process and pharmaceutical intervention

### 3.1 Initial treatment regimen

On the first day of admission: The patient was transferred to the RICU due to respiratory failure. Endotracheal intubation, nasogastric tube placement, and urinary catheterization were performed immediately upon admission. The patient’s maximum body temperature reached 38.9°C on the same day. Arterial blood gas analysis results were as follows: pH 7.40, partial pressure of carbon dioxide (PaCO_2_) 36.2 mmHg, partial pressure of oxygen (PaO_2_) 102 mmHg. Electrocardiographic monitoring showed BP 110/55 mmHg, HR 98 beats/min, peripheral oxygen saturation (SpO_2_) 96%, and RR 18 breaths/min. Laboratory test results revealed elevated indicators: white blood cell count (WBC) 10.34×10^9^/L↑, neutrophil percentage (NE%) 89.5%↑, CRP 200.64 mg/L↑, PCT 1.15 ng/mL↑, CK 5127 U/L↑, ALT 271 U/L↑, AST 417 U/L↑, and estimated glomerular filtration rate (eGFR) 50 mL/(min/1.73 m²). Given the patient’s acute respiratory distress syndrome (ARDS), rapidly progressive pulmonary lesions, and elevated muscle enzyme levels, a diagnosis of suspected *Legionella* infection was considered. The treatment regimen was formulated as follows: Anti-infective therapy: Meropenem for injection 1 g, intravenous drip, every 8 hours; moxifloxacin hydrochloride and sodium chloride injection 0.4 g, intravenous drip, once daily at night. Hepatoprotective therapy: Glutathione for injection 1.8 g, intravenous drip, once daily; magnesium isoglycyrrhizinate injection 200 mg, intravenous drip, once daily. Interventions for elevated CK: Hydration with 0.9% sodium chloride injection; alkalization of urine with sodium bicarbonate tablets.

### 3.2 Disease progression and regimen adjustment

On the second day of admission: The patient still presented with fever (maximum temperature 39.2°C). Arterial blood gas analysis results were: pH 7.45, PaCO_2_ 36 mmHg, PaO_2_ 77 mmHg. Laboratory tests showed CRP 162 mg/L↑ and PCT 1.13 ng/mL↑. Considering the rapidly progressing pulmonary lesions combined with muscle injury and the positive influenza A nucleic acid test result from the external hospital, viral infection could not be ruled out. The treatment regimen was adjusted by adding anti-influenza agents and anti-inflammatory and antithrombotic drugs: Baloxavir marboxil tablets 20 mg, oral administration, single dose; peramivir sodium chloride injection 300 mg, intravenous drip, once daily. Methylprednisolone sodium succinate for injection 40 mg, intravenous drip, once daily (anti-inflammatory therapy). Enoxaparin sodium injection 4000 AxaIU, subcutaneous injection, once daily (thromboprophylaxis). Pharmaceutical recommendations: Given the patient’s rhabdomyolysis combined with hepatorenal dysfunction, it was recommended to monitor renal function closely and adjust the dosage of meropenem according to creatinine clearance rate. Additionally, moxifloxacin might exacerbate muscle injury, so close monitoring of CK levels was advised. Clinical decision: The original anti-infective regimen was temporarily maintained; close monitoring of renal function and CK levels was implemented, and symptomatic supportive care was strengthened.

On the third day of admission: The patient was conscious and afebrile, with intermittent productive cough of copious yellowish-white viscous sputum. Pulmonary rales were not completely resolved, and SpO_2_ remained above 95%. Endotracheal extubation was performed on this day. Arterial blood gas analysis results were: pH 7.49, PaCO_2_ 32.9 mmHg, PaO_2_ 71 mmHg. Physical examination findings were: temperature 37.3°C, HR 79 beats/min, RR 22 breaths/min, BP 160/68 mmHg. Laboratory tests showed WBC 9.86×10^9^/L↑, NE% 87.74%↑, CRP 158 mg/L↑, PCT 0.58 ng/mL↑, ALT 165 U/L↑, AST 141 U/L↑, CK 679 U/L↑, and serum creatinine 201.5 μmol/L. The results of tNGS of bronchoalveolar lavage fluid (BALF) were available, indicating *Cps* infection with 7579 sequence reads and a relative abundance of 94.9% (**Table 1**),

**Table 1:** Results of tNGS of the patient’s BALF.

Class	Pathogen	Sequence number	Relative abundance
Bacteria	*Chlamydia psittaci*	7579	94.9%
Fungi	–	–	–
Virus	Rhinovirus type A	18	19.5%
Parasite	–	–	–
Human microorganisms	Human herpesvirus 4	10	0.5%
	Human herpesvirus 7	3	0.2%

“-” means not detected. Only pathogens/microorganisms identified as positive by bioinformatics analysis are listed. Relative abundance represents the percentage of sequence reads of a given pathogen out of the total sequence reads of all detected pathogens.

Detailed tNGS methodology is provided in the Supplementary Materials (**Supplementary Material 1**). A definitive diagnosis of severe *Cps* pneumonia was thus confirmed. Further inquiry about the epidemiological history revealed that the patient and his family had no history of parrot breeding or exposure to poultry. Neither influenza nucleic acid testing nor tNGS detected influenza A virus, so peramivir was discontinued. Persistently elevated liver enzyme levels were considered to be secondary to rhabdomyolysis caused by severe infection.

Pharmaceutical Recommendations: According to clinical guidelines, tetracyclines are the first-line agents for *Cps* infection. Given the patient’s severe infection, moxifloxacin could be continued and combined with doxycycline 0.1 g orally twice daily for targeted therapy, with a treatment course of at least 10 days. In view of the patient’s renal insufficiency, dose adjustment of meropenem was recommended; the drug should be continued until infection-related biomarkers improved significantly to cover potential concurrent bacterial infections. Clinical Protocol: Starting on the same day, doxycycline tablets 0.1 g were administered via nasogastric tube twice daily. The dosage of meropenem was adjusted to intravenous infusion every 12 hours based on the patient’s renal function, and both meropenem and moxifloxacin were continued.

On the 4th day of admission: The patient was conscious and afebrile, with intact swallowing function and normal heart sounds. Auscultation of the chest revealed scattered wheezes in both lungs. The nasogastric tube was removed on this day, and oral feeding was resumed. Arterial blood gas analysis results: pH 7.55, PaCO_2_ 64.6 mmHg, PaO_2_ 66 mmHg. Physical examination findings: temperature 37.3°C, HR 115 beats/min, RR 22 breaths/min, BP 129/61 mmHg. Laboratory test results: WBC 13.34×10^9^/L↑, NE% 82.73%↑, CRP 92.3 mg/L↑, PCT 0.24 ng/mL, CK 430 U/L↑, ALT 166 U/L↑, AST 102 U/L↑, eGFR 35.36 mL/(min/1.73 m²). Clinical Protocol: Doxycycline administration was switched from nasogastric tube to oral route.

On the 5th day of admission: The patient remained conscious with low oxygen requirements. Infection-related biomarkers, CK and myoglobin levels showed a progressive decline. The patient was planned to be transferred to the general ward for further treatment on this day. Physical examination findings: temperature 37.3°C, HR 59 beats/min, RR 22 breaths/min, BP 144/90 mmHg. Laboratory test results: WBC 12.02×10^9^/L, NE% 80.3%, CRP 48.3 mg/L, PCT 0.09 ng/mL, CK 213 U/L↑, ALT 161 U/L↑, AST 214 U/L↑, eGFR 44.94 mL/(min/1.73 m²).

From the 6th to the 11th day of admission: Starting on the 6th day, the anti-infective regimen was modified to monotherapy with doxycycline tablets 0.1 g orally twice daily. Adjuvant therapies including hepatoprotection, nephroprotection and anticoagulation were continued. Subsequently, the patient’s cough and expectoration symptoms alleviated, and liver and renal function parameters improved gradually. CK decreased to 197 U/L and CRP dropped to 13.81 mg/L. Chest CT scan on December 10, 2025 showed that left-sided pleural effusion and the scope of consolidation in the left lower lung lobe were reduced compared with prior images (**Figure 1E**). The patient’s condition stabilized and he was discharged after 11 days of hospitalization. Discharge medications: Doxycycline 100 mg orally twice daily (to continue anti-infective therapy, with a total course of 14 days).

## 4 Treatment outcomes and follow-up results

**Figure 2:**
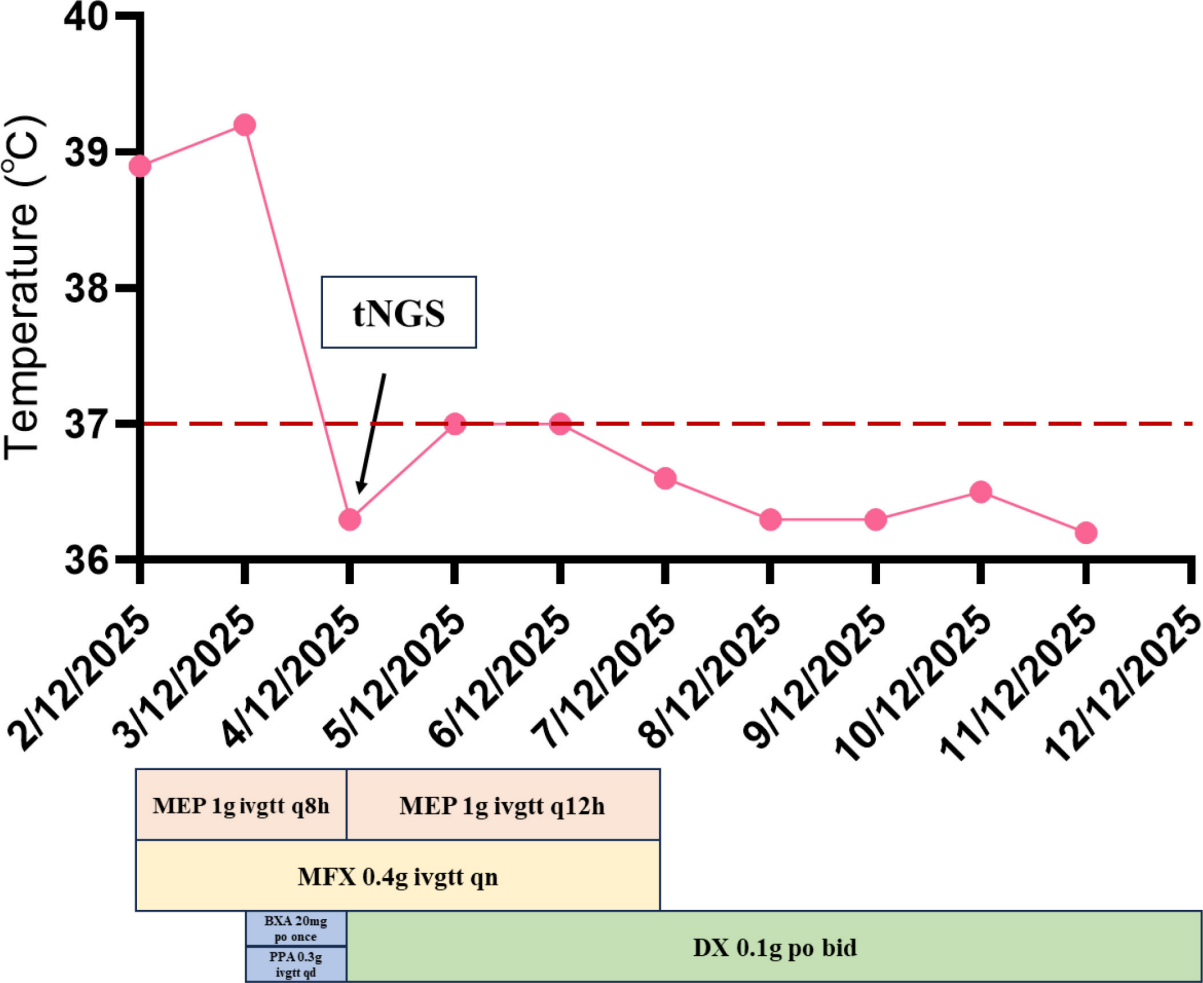
Temperature fluctuations and anti-infective medication administration during the patient’s hospitalization. MEP, Meropenem; MFX, Moxifloxacin; DX, Doxycycline; BXA, Baloxavir; PPA, Peramivir.

**Figure 3:**
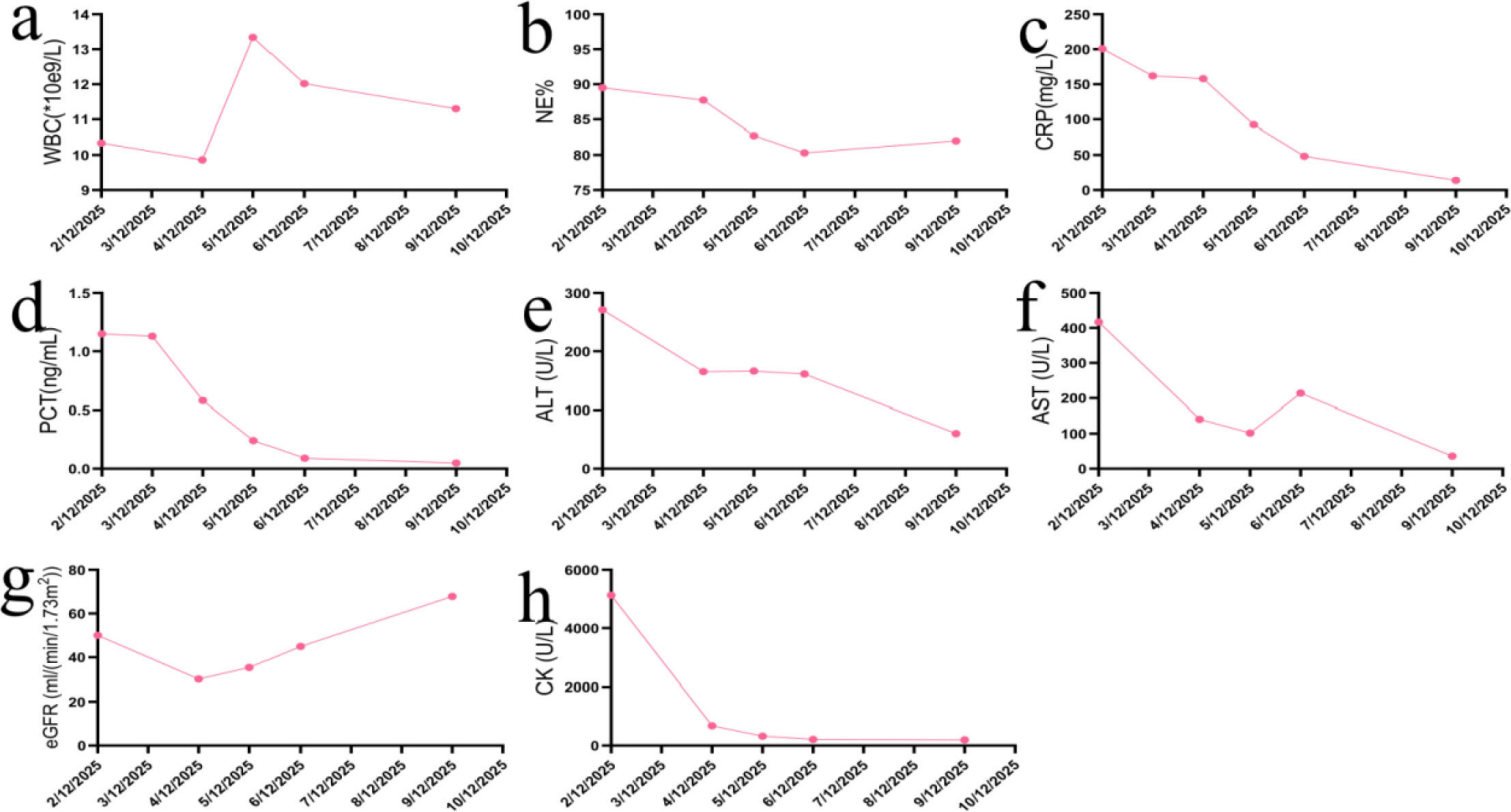
Changes in major blood test indicators during the patient’s disease course. **(a)** WBC; **(b)** NE%; **(c)** CRP; **(d)** PCT; **(e)** ALT; **(f)** AST; **(g)** CK; **(h)** eGFR. Normal reference ranges: WBC 3.5-9.5×10^9^/L; NE% 40-75%; CRP 0–4 mg/L; PCT <0.05 ng/mL; ALT <40 U/L; AST <40 U/L; CK <200 U/L; eGFR >90 mL/min/1.73m².

The patient’s fever resolved starting from December 4, 2025, with improved mental status and alleviated symptoms of dyspnea and chest tightness (**Figure 2**). On the basis of anti-infective therapy administered in the RICU, doxycycline hydrochloride tablets were added for 3 days. Consequently, rapid improvements were observed in infection-related biomarkers, liver and renal function parameters, and CK levels. The patient was then transferred to the general ward and switched to doxycycline monotherapy. After 5 days of monotherapy, the patient’s symptoms were relieved, and he was successfully discharged from the hospital (**Figure 3**). At the follow-up conducted two weeks after discharge, the patient had completed the full course of doxycycline treatment. A follow-up chest CT scan was not performed temporarily, and the patient reported no discomfort.

## 5 Literature review

Studies have shown that compared with doxycycline and azithromycin, moxifloxacin offers no advantages in terms of administration regimen, treatment course, or disease prognosis when used for the treatment of *Cps* infection (Fraeyman et al., 2010). Particularly in patients with severe infections, its use may even increase the risk of mortality. Therefore, fluoroquinolones are not recommended as the first-line treatment for *Cps* infection. We searched PubMed for case reports on moxifloxacin therapy for *Cps* pneumonia published in the past 20 years, due to the heterogeneity of case definitions, severity, and adjunct therapies, a formal meta-analysis was not performed, and outcomes are described narratively. (**Table 2**). The results identified 17 cases treated with moxifloxacin, among which 10 cases achieved clinical efficacy and 7 cases showed no response. Specifically, moxifloxacin was used as monotherapy in 10 cases, with 4 cases responded effectively and 6 cases failed; moxifloxacin was combined with doxycycline in 6 cases, with 5 cases responded effectively and 1 case failed; 1 case received moxifloxacin combined with omadacycline. The median treatment course during hospitalization was 7.5 (interquartile range, 4.5–14.0) days. Overall, favorable clinical responses were reported in approximately half of the cases, but outcomes varied widely. No direct comparison of efficacy between different regimens can be made from these heterogeneous reports. Importantly, several cases that failed on moxifloxacin (either alone or in combination) subsequently improved after switching to a tetracycline-class antibiotic (doxycycline or omadacycline). This observation supports the current guideline recommendation that tetracyclines remain the first-line therapy for *Cps*, while fluoroquinolones should be reserved for specific circumstances (e.g., contraindications or intolerance to tetracyclines).

**Table 2:** Cases summary of moxifloxacin in the treatment of *Cps* pneumonia.

Number	Therapeutic medication	Length of stay (days)	Medication and course of treatment after discharge	Prognosis	Notes	Ref
1	Moxifloxacin + Doxycycline	21	Doxycycline for 7 days	Cure	–	(Fang et al., 2023)
2	Moxifloxacin + Doxycycline	26	None	Cure	–	(Fang et al., 2022b)
3	Moxifloxacin + Doxycycline	8 + 7	None	Improvement	–	(He et al., 2023)
4	Moxifloxacin	14	Moxifloxacin for 1 week + clarithromycin for another 1 month+ methylprednisolone for 2 weeks	Cure	–	(Gao et al., 2022)
5	Moxifloxacin	3	None	Fail	Improvement after doxycycline treatment	(Yang et al., 2023)
6	Moxifloxacin	3	None	Fail	Moxifloxacin failed to be treated and was replaced with omadacycline, which improved and discharged	(Fang et al., 2022c)
7	Moxifloxacin	3	None	Fail	The conversion to doxycycline + piperacillin tazobactam still failed, and finally omadacycline was successfully cured.	(Wang et al., 2024a)
8	Moxifloxacin	3	None	Fail	The conversion to doxycycline still failed, and finally omadacycline was successfully cured.	(Xu and Huang, 2025)
9	Moxifloxacin + Doxycycline	10	None	Cure	–	(Li et al., 2023)
10	Moxifloxacin	7	Moxifloxacin for 1 week	Fail	Improvement after treatment with tetracycline	(Li et al., 2025)
11	Moxifloxacin + Doxycycline	6 + 3	Unknown	Cure	–	(Wu et al., 2024)
12	Moxifloxacin	7	Moxifloxacin for 1 week	Cure	–	(Zhang et al., 2024b)
13	Moxifloxacin	14	None	Cure	–	(Zhang et al., 2024a)
14	Moxifloxacin	14	None	Cure	–	(Guillemot et al., 2025)
15	Moxifloxacin + Doxycycline	5	None	Fail	Improvement after treatment with Omadacycline	(Yang et al., 2025)
16	Moxifloxacin + Omadacycline	10	None	Improvement	Sequential therapy with oral doxycycline	(Gao et al., 2025)
17	Moxifloxacin	Unknown	None	Fail	Improvement after treatment with azithromycin	(Vande Weygaerde et al., 2018)

- stands for “None”. Improvement: Clinical stabilization and discharge; Cure: Completion of therapy with symptom resolution; Fail: Required change of antibiotic class due to lack of response. Follow-up durations varied across reports but generally ranged from 2 weeks to 1 month post-discharge.

## 6 Analysis and discussion

### 6.1 Clinical characteristics and diagnosis of severe *Cps* Pneumonia

*Cps* is a Gram-negative obligate intracellular pathogen, with birds serving as the primary reservoir. Human infections are mainly transmitted through contact with infected avian species, and the clinical manifestations are dominated by influenza-like symptoms such as high fever and cough. Studies have shown that even mild initial infections may lead to severe long-term sequelae (Deng et al., 2024). Severe cases can involve multiple organs, presenting with complications including respiratory failure, rhabdomyolysis, and hepatorenal dysfunction (Shi et al., 2021; Deng et al., 2024). In addition to pulmonary infection, the patient in this case was complicated with acute respiratory failure, rhabdomyolysis, hepatorenal dysfunction and lower extremity venous thrombosis, which are consistent with the severe manifestations of critical cases. Clinicians should conduct detailed medical history inquiries to improve the efficacy of initial empirical anti-infective regimens. Notably, the patient and his family denied any history of parrot contact or poultry breeding, suggesting that the infection might have been acquired via inhalation of contaminated airborne droplets.

Conventional detection methods exhibit low sensitivity for *Cps*. NGS technology, with its advantages of high throughput and rapid identification, has emerged as a vital tool for diagnosing rare pathogen infections. In particular, tNGS further enhances the detection rate of rare pathogens. The definitive diagnosis of this patient via tNGS provided critical evidence for subsequent targeted therapy, indicating that tNGS should be performed as early as possible to identify the pathogen in patients with severe pneumonia unresponsive to conventional treatment. In this test, only 18 specific sequences of rhinovirus A were detected, which was far below the typical threshold for clinically active infection (usually requiring >100 reads for clinical significance). Considering that the patient had no significant upper respiratory tract cataract symptoms, we determined that he was an asymptomatic carrier or had respiratory tract colonization, and no targeted antiviral treatment was required. Similarly, the detection of EBV and HHV-7 (10 and 3 sequences respectively) in BALF samples usually reflects the reactivation of latent viruses in alveolar macrophages or the trace incorporation of oral colonization, and is not the pathogenic pathogen of this pneumonia.

*Cps* infections display strong clinical phenotypic heterogeneity. Critical cases are often associated with diagnostic delays due to the absence of specific symptoms and clear exposure history (Hogerwerf et al., 2017; McGovern et al., 2021). Breaking away from the traditional diagnostic framework of “exposure history plus symptoms”, this case achieved the dual goals of “accurate diagnosis and differential diagnosis” via tNGS, despite initial suspicions of *Legionella* and influenza virus infection with nonresponsive treatment outcomes. This technique not only rapidly identified *Cps* as the pathogenic agent but also ruled out influenza A virus, which had tested positive in previous assays, thereby correcting the initial diagnostic bias. Existing studies have mostly emphasized the value of tNGS in detecting rare pathogens (Chen et al., 2024; Yin et al., 2024; Kristan and Debeljak, 2025). This case further confirms that for complex critical cases with multiple organ damage, tNGS can shorten the diagnostic cycle (only 3 days from admission to definitive diagnosis in this case) through “precise etiological localization and exclusion of nontarget pathogens”, thereby gaining valuable time for subsequent targeted therapy. Additionally, subsequent inquiries confirmed that the patient had no history of parrot ownership, further suggesting that for patients with severe CAP without clear exposure history and negative results from conventional tests, clinicians should correlate the findings with tNGS results to avoid missed diagnosis of occult infections.

### 6.2 Optimization of anti-infective treatment regimen

*Cps* is insensitive to β-lactam antibiotics. Tetracyclines (e.g., doxycycline) are first−line, with a recommended treatment duration of 14 days in general, and some guidelines also suggest a course of 10–21 days. Macrolides are alternatives when tetracyclines are contraindicated (Stewardson and Grayson, 2010). However, with the widespread clinical use of these agents, the rates of resistance to tetracyclines and macrolides have increased significantly in recent years (Pawlikowska-Warych et al., 2015; Li et al., 2017; Benamri et al., 2021). Fluoroquinolones are also active against Chlamydia species, and moxifloxacin has shown efficacy in some case reports (Guillemot et al., 2025).

In this case, the initial anti-infective regimen consisted of meropenem combined with moxifloxacin, which could cover common pathogens of CAP as well as atypical pathogens such as *Legionella* species. Nevertheless, the patient’s clinical symptoms failed to improve after empirical therapy. After tNGS confirmed *Cps* infection, doxycycline was added. The combination of doxycycline and moxifloxacin has demonstrated favorable effects against Chlamydia pneumoniae (Fang et al., 2022a). Mechanistically, fluoroquinolones inhibit DNA replication, while tetracyclines block protein synthesis, their distinct targets may produce synergistic or additive effects (Beeckman and Vanrompay, 2009; Gu et al., 2020; Wang et al., 2024b). For severe Cps infections with multi−organ dysfunction, combination therapy is supported by clinical practice, though treatment must be individualized.

Given the patient’s multi−organ dysfunction, clinical pharmacists prioritized drug safety. Doxycycline has good oral absorption and does not require renal dose adjustment. The elevated liver enzymes were considered secondary to severe infection−induced rhabdomyolysis, so infection control was the core goal. Thus, doxycycline was appropriate. Although no other pathogens were identified, studies report that 38.7% of psittacosis patients have concurrent bacterial infections (Donati et al., 1999). Therefore, meropenem was continued until infection biomarkers improved, covering potential mixed infections.

Current guidelines recommend tetracyclines as first-line therapy for *Cps* infection (Stewardson and Grayson, 2010). For this critically ill patient with delayed diagnosis and multi−organ failure, we considered doxycycline plus moxifloxacin justified. However, this should not be generalized. Tetracycline monotherapy remains the standard for less severe cases, and our findings are preliminary, requiring validation in larger studies.

### 6.3 Key points of pharmaceutical care

Organ Function Monitoring: The patient had rhabdomyolysis and hepatorenal dysfunction. We regularly monitored CK, liver enzymes, and renal function, and adjusted drug dosages promptly to avoid additional organ burden. Adverse Drug Reaction Monitoring: Doxycycline may cause gastrointestinal and photosensitivity reactions; the patient was advised to take it after meals and avoid sun exposure. For meropenem, renal function guided dosing intervals. Moxifloxacin was used with close observation for muscle pain; it would be discontinued if needed to prevent worsening of rhabdomyolysis. Patient Education: At discharge, the patient was instructed to complete the 14-day doxycycline course, not to stop medication without advice, and to attend follow-up (chest CT, liver/renal tests, lower-extremity ultrasound). He was also told to seek immediate care if fever or cough recurred.

Full-course involvement of clinical pharmacists was key to success. We applied a full−cycle pharmaceutical intervention model: precise drug selection, dosage adjustment, safety monitoring, and patient education. Specifically: (1) Drug selection-doxycycline was chosen because it does not require renal adjustment. (2) Intra-treatment monitoring-CK was tracked continuously (dropping from >20640 U/L to 197 U/L), liver and renal function were monitored, and hepatoprotective agents were adjusted. (3) Discharge education–the patient was instructed on post−meal intake and sun protection to ensure adherence. Most existing case reports have focused primarily on clinical treatment outcomes (Shi et al., 2021; Zhang et al., 2022). In contrast, this case highlights the professional value of clinical pharmacists in the management of severe *Cps* infection, an aspect that has been less emphasized in previous reports. Through multidisciplinary collaboration (respiratory medicine plus clinical pharmacy), the balance between therapeutic efficacy and safety was achieved, providing a practical reference for pharmaceutical intervention in complex severe infections.

## 7 Conclusion

Severe *Cps* pneumonia is prone to complicated with multiple organ dysfunction. For clinical diagnosis, it is necessary to combine the patient’s history of avian exposure and typical clinical manifestations, and adopt precise detection techniques such as NGS to identify the pathogen, so as to avoid missed diagnosis and misdiagnosis. Targeting this case of severe *Cps* pneumonia characterized by “occult infection + complex complications + failure of empirical therapy”, we provides a practical reference for the management of similar rare severe infections, particularly highlighting the utility of tNGS in cases lacking clear exposure history and the importance of clinical pharmacy involvement. However, given the inherent limitations of a single case report, these findings should be interpreted as hypothesis-generating rather than practice-defining. The clinical implications are as follows: (1) For patients with severe CAP complicated with multiple organ damage, if initial empirical therapy fails within 48–72 hours, tNGS testing should be initiated as early as possible to avoid missed diagnosis of rare pathogens; (2) For severe *Cps* infection combined with multiple organ dysfunction, combination therapy with tetracyclines plus fluoroquinolones is recommended, with dynamic dosage adjustment based on the status of organ function; (3) Clinical pharmacists should be deeply involved in the treatment decision-making of complex severe cases, providing full-process support ranging from drug selection and dosage optimization to adverse reaction monitoring, so as to reduce treatment risks. The diagnosis and treatment experience of this case provides new insights for the management of similar rare severe infections, and also offers practical reference for the application of multidisciplinary collaboration in severe infectious diseases. It should be clarified that this study is a single case report with a limited sample size. The clinical characteristics, diagnosis and treatment process, and prognosis of the case may have individual specificity. Further retrospective or prospective studies with large sample sizes are required to verify the relevant treatment regimens.

## Data Availability

The original contributions presented in the study are included in the article/**Supplementary Material**. Further inquiries can be directed to the corresponding author.
