# Supplementary material for: Clinical management and pharmaceutical care of severe Chlamydia psittaci Pneumonia complicated with rhabdomyolysis and multiple organ dysfunction: a case report and literature review
Source: Front Cell Infect Microbiol. 2026 May 13;16:1785697. doi: 10.3389/fcimb.2026.1785697 (PMC13212441; doi:10.3389/fcimb.2026.1785697)
Supplement: Supplementary file 1 [file Table1.docx]

****tNGS Methodology****

Bronchoalveolar lavage fluid (BALF) was collected from the patient on day 2 of admission. Targeted next-generation sequencing (tNGS) was performed using the IDseq Focus respiratory tract 300+ panel (Vision Medicals, China), which covers 133 bacteria, 46 fungi, 110 DNA/RNA viruses, 13 parasites, and 35 key resistance/virulence genes. Library preparation, probe capture, and sequencing were conducted following the manufacturer‘s protocol. Sequencing was performed on an Illumina NextSeq CN500 platform with single-end 75 bp sequencing, generating approximately 20 million reads per library. A negative control (NC) was included in each run, and the NC result was qualified, indicating no significant contamination.

Host nucleic acid removal, DNA/RNA extraction, and bioinformatics analysis :

Pathogens and human cells were separated from 1 mL BALF samples by centrifuging it at 12,000 g for 5 min. The host nucleic acid was then removed from the precipitate using 1 U Benzonase (Sigma) and 0.5% Tween 20 (Sigma), which were incubated at 37°C for 5 min. A 400 µL dose of terminal buffer was then added to halt the reaction. A Minilys Personal TGrinder H24 Homogenizer (catalog number: OSE-TH-01, Tiangen, China) was used to beat beads after transferring a total of 600 µl mixture into new tubes containing 500 µl of ceramic beads. DNA was then extracted and eluted from 400 µL of pretreatment samples using a QIAamp UCP Pathogen Mini Kit in 60 µL elution buffer (catalog number: 50214, Qiagen, Germany). Using a Qubit dsDNA HS Assay Kit (catalog number: Q32854, Invitrogen, USA), the isolated DNA was quantified. Total RNA was extracted with a QIAamp® Viral RNA Kit (Qiagen) and ribosomal RNA was removed by a Ribo-Zero rRNA Removal Kit (Illumina). cDNA was generated using reverse transcriptase and dNTPs (Thermo Fisher). Libraries were constructed for the DNA and cDNA samples using the KAPA low throughput library construction kit (KAPA Biosystems, U.S.A.) following the manufacturer's instructions. Library was quality assessed by Qubit dsDNA HS Assay kit followed by High Sensitivity DNA kit (Agilent) on an Agilent 2100 Bioanalyzer. Library pools were then loaded onto an Illumina Nextseq CN500 sequencer for 75 cycles of single-end sequencing to generate approximately 20 million reads for each library.
